# Complex Mixture Analysis of Organic Compounds in Yogurt by NMR Spectroscopy

**DOI:** 10.3390/metabo6020019

**Published:** 2016-06-16

**Authors:** Yi Lu, Fangyu Hu, Takuya Miyakawa, Masaru Tanokura

**Affiliations:** Department of Applied Biological Chemistry, Graduate School of Agricultural and Life Sciences, The University of Tokyo, 1-1-1 Yayoi, Bunkyo-ku, Tokyo 113-8657, Japan; ly22999@hotmail.com (Y.L.); fangyu_hu@hotmail.com (F.H.); atmiya@mail.ecc.u-tokyo.ac.jp (T.M.)

**Keywords:** NMR, assignment, yogurt, complex mixture analysis, existing state, DOSY, quantification

## Abstract

NMR measurements do not require separation and chemical modification of samples and therefore rapidly and directly provide non-targeted information on chemical components in complex mixtures. In this study, one-dimensional (^1^H, ^13^C, and ^31^P) and two-dimensional (^1^H-^13^C and ^1^H-^31^P) NMR spectroscopy were conducted to analyze yogurt without any pretreatment. ^1^H, ^13^C, and ^31^P NMR signals were assigned to 10 types of compounds. The signals of α/β-lactose and α/β-galactose were separately observed in the ^1^H NMR spectra. In addition, the signals from the acyl chains of milk fats were also successfully identified but overlapped with many other signals. Quantitative difference spectra were obtained by subtracting the diffusion ordered spectroscopy (DOSY) spectra from the quantitative ^1^H NMR spectra. This method allowed us to eliminate interference on the overlaps; therefore, the correct intensities of signals overlapped with those from the acyl chains of milk fat could be determined directly without separation. Moreover, the ^1^H-^31^P HMBC spectra revealed for the first time that *N*-acetyl-d-glucosamine-1-phosphate is contained in yogurt.

## 1. Introduction

NMR spectroscopy is highly quantitative and reproducible, and its sensitivity does not depend on the types of metabolites [1]. As a non-targeted method, NMR measurements do not require separation and chemical modification; therefore, comprehensive information regarding the chemical components of mixtures can be rapidly and directly provided [2,3]. For the past decade, NMR has been recognized as a powerful technique for discerning the chemical properties of complex mixtures and has been widely applied to identify organic compounds in foods, such as milk [4], soy sauce [5], coffee [6], wine [7], mango juice [8], and green tea [9]. Among NMR-based comprehensive analyses, ^1^H NMR is the most useful tool because of its informative spectral patterns and high-throughput acquisition. Compared with the NMR signals of isolated compounds, chemical shift changes are often caused by interactions with other compounds in food mixtures [10,11]. In addition, food mixtures can also lead to extreme signal overlaps. Therefore, ^13^C NMR spectra and two-dimensional NMR spectroscopy are necessary to help assign each NMR signal in complex food mixtures.

Yogurt is traditionally made from milk fermentation by thermophilic cultures of *Lactobacillus* *delbrueckii* subsp. *bulgaricus* and *Streptococcus thermophilus*. *L.* *delbrueckii* subsp. *bulgaricus* metabolizes sugar to lactic acid with small amounts of by-products [12]. The benefits of yogurt include lactose digestion, intestinal microflora modulation, cholesterol reduction, immune system stimulation, and cancer prevention [13]. Texture is also a factor that influences the quality of yogurt and is related to sensory perception of food products. Therefore, the chemical properties of yogurt can help us analyze and improve the functional and gustatory qualities of yogurt [14].

Many yogurt studies have been dedicated to identifying the chemical compositions of yogurt [15,16,17]. In these studies, organic acids, acetaldehyde, and other compounds in yogurt have been analyzed with conventional techniques, such as GC-MS [16] and LC-MS [17]. These methods require appropriate extraction, separation and chemical derivatization of individual components. However, qualitative and quantitative modifications of the original mixture can even be performed by a simple treatment without any separation. A method for the simultaneous and nondestructive identification of compounds in yogurt has not been established.

In a previous study, we reported the signals assignments of NMR spectra of milk, and the analysis of the existing states of components [10]. Here, we assigned the signals of yogurt by combining 1D and 2D NMR spectroscopy without changing the chemical compositions. In addition, quantitative difference spectra between quantitative ^1^H NMR spectra and diffusion ordered spectroscopy (DOSY) spectra were applied to quantitatively analyze the fermented metabolites whose signals overlapped with broad signals from milk fat.

## 2. Results and Discussion

### 2.1. ^1^H NMR Spectra of Yogurt

**Figure 1:**
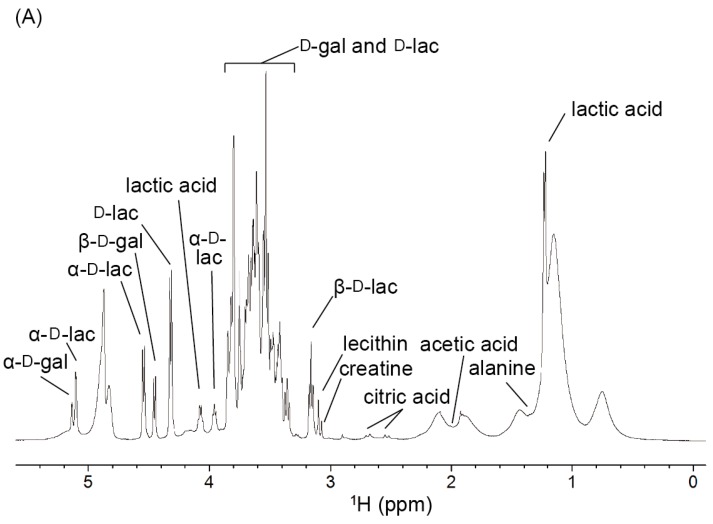

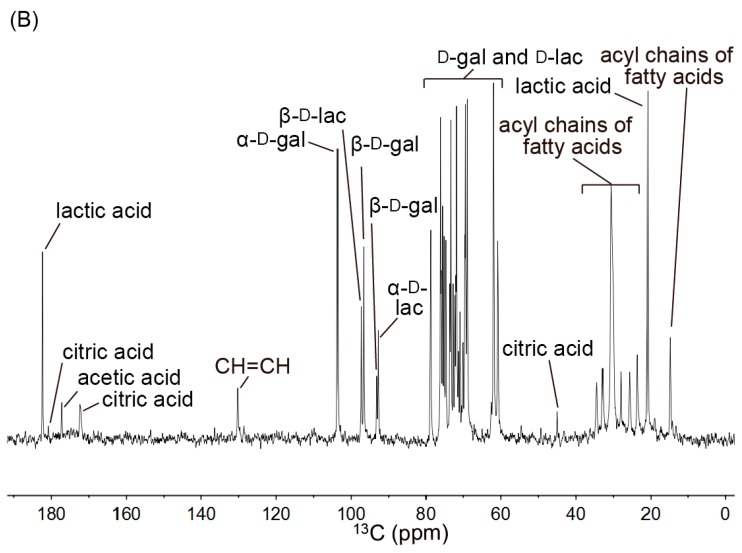
(**A**) The ^1^H NMR spectrum of yogurt. (**B**) The ^13^C NMR spectrum of yogurt. Gal and lac are abbreviations for galactose and lactose, respectively.

A typical ^1^H NMR spectrum of yogurt is shown in Figure 1A. Proton resonances of aliphatic groups were observed with considerable overlap in the region from 0.6 to 2.2 ppm. These signals were assigned to the acyl chains of fatty acids from milk fats. In addition, the signals at 1.23 and 1.30 ppm were assigned to lactic acid and alanine, respectively. Compared with the chemical shifts on the database, the weak signals from 2.2 to 3.1 ppm were suggested to be methyl or methylene groups of citrate, creatine, and lecithin, which are minor components of yogurt. These signals were finally assigned by interpreting the cross peaks on the ^1^H-^13^C HSQC, ^1^H-^1^H DQF-COSY, and ^1^H-^13^C CT-HMBC spectra. The signals from 3.1 to 5.1 ppm were assigned to d-lactose and D-galactose. These signals were sharp and highly sensitive but heavily overlapped with one another. Therefore, their precise assignments were achieved by the ^13^C NMR and ^1^H-^13^C HSQC spectra. However, the region from 5.5 to 9.0 ppm contained several weak signals that were considered to be the amide protons of proteins. Caseins and lactoglobulins are the major proteins of cow's milk [18,19]. These proteins and their degradation products may be observed in the ^1^H NMR spectra of yogurt. The details of the signal assignments are summarized in Table 1.

### 2.2. ^13^C NMR Spectra of Yogurt

A typical ^13^C NMR spectrum of yogurt is shown in Figure 1B. The signals were well separated and were completely assigned by utilizing the ^1^H-^13^C HSQC, ^1^H-^1^H DQF-COSY, and ^1^H-^13^C CT-HMBC spectra. The signals from 10 to 55 ppm were assigned to the primary carbon atoms from the acyl chains of fatty acids, lactic acid, and acetic acid. This region also contained some signals from minor components, which were assigned to be alanine, creatine, and citrate. There were signals of tertiary carbon atoms from the aliphatic rings of lactose and galactose in the region from 55 to 110 ppm. The signals from 160 to 190 ppm were assigned to the quaternary carbon atoms of organic acids and alanine based on the ^1^H-^13^C CT-HMBC spectrum. The details of the signal assignments are summarized in Table 1.

### 2.3. Identification of Several Components with 2D NMR Spectra

**Figure 2:**
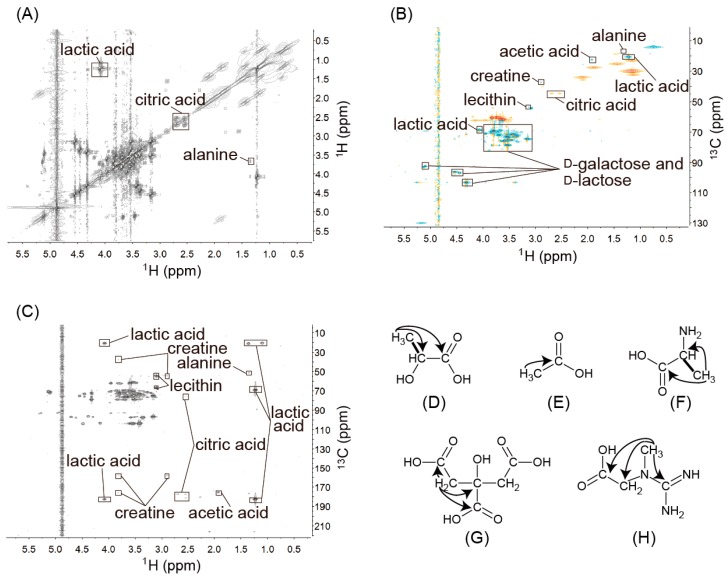
(**A**) The ^1^H–^1^H COSY NMR spectrum of yogurt. (**B**) The ^1^H–^13^C HSQC NMR spectrum of yogurt. (**C**) The ^1^H–^13^C HMBC NMR spectrum of yogurt. (**D**‒**H**) Correlations of lactic acid (**D**), acetic acid (**E**), alanine (**F**), citrate (**G**), and creatine (**H**). Arrows show the cross-peaks in the ^1^H–^13^C HMBC NMR spectrum, and bold bonds show the cross-peaks in the ^1^H–^1^H COSY NMR spectrum. Gal and lac are abbreviations for galactose and lactose, respectively.

*Lactic acid*. In the ^1^H-^1^H DQF-COSY spectra of yogurt (Figure 2A), the cross-peaks at 1.23 and 4.08 ppm were the correlations between –CH_3_ and –CH (Figure 2D), while the ^1^H signals at 1.23 ppm showed correlations with the ^13^C signals at 20.84 ppm, and the ^1^H signals at 4.08 ppm showed correlations with the ^13^C signals at 68.95 ppm in the HSQC spectrum (Figure 2B). The ^1^H signals at 1.23 and 4.08 ppm were also connected to the ^13^C signals at 182.41 ppm in the ^1^H-^13^C CT-HMBC spectrum (Figure 2C,D), which indicated correlations of –CH_3_ and –CH with –COOH.

*Acetic acid*. The ^1^H signals at 1.93 ppm showed correlations with the ^13^C signals at 22.72 ppm in the HSQC spectrum (Figure 2B) and were connected to the ^13^C signals at 175.46 ppm in the ^1^H-^13^C CT-HMBC spectrum (Figure 2C). Therefore, the correlation between –CH_3_, and –COOH of acetic acid was confirmed (Figure 2E).

*Alanine*. In the ^1^H-^1^H DQF-COSY spectra of yogurt (Figure 2A), the cross peaks at 1.35 and 3.65 ppm were assigned to the correlations between –CH_3_ and –CH. In the ^1^H-^13^C CT-HMBC spectrum (Figure 2C), the ^1^H signals at 1.35 and 3.65 ppm showed correlations with the ^13^C signals at 176.40 ppm, which confirmed the connectivity among –CH_3_, –CH, and –COOH of alanine (Figure 2F).

*Citrate*. The ^1^H signals at 2.53 and 2.69 ppm showed correlations with the ^13^C signals at 44.93 ppm in the HSQC spectrum and were also connected to the ^13^C signals at 75.90, 177.18, and 180.81 ppm (Figure 2C,G) in the ^1^H-^13^C CT-HMBC spectrum, which suggested that the signals should be assigned to citrate. In whole milk, the citrate signals were assigned as the ^1^H signals at 2.40 and 2.54 ppm, and the ^13^C signals at 44.93 ppm [4]. Citrate is the main organic acid in milk and binds to calcium ions to act as a component of casein micelles [20]. The variety of chemical shifts may reflect differences in the existing states of casein micelles, pH, and temperature.

*Creatine*. In the ^1^H-^13^C CT-HMBC spectrum (Figure 2C), the ^1^H signals at 3.81 ppm were connected to the ^13^C signals at 37.40, 158.30, and 175.48 ppm, while the ^1^H signals at 2.90 ppm showed correlations with the ^13^C signals at 54.78 and 158.30 ppm (Figure 2C,H), which indicated correlations of –CH_3_ with –CH_2_, –C[(NH)NH_2_], and –COOH.

**Figure 3:**
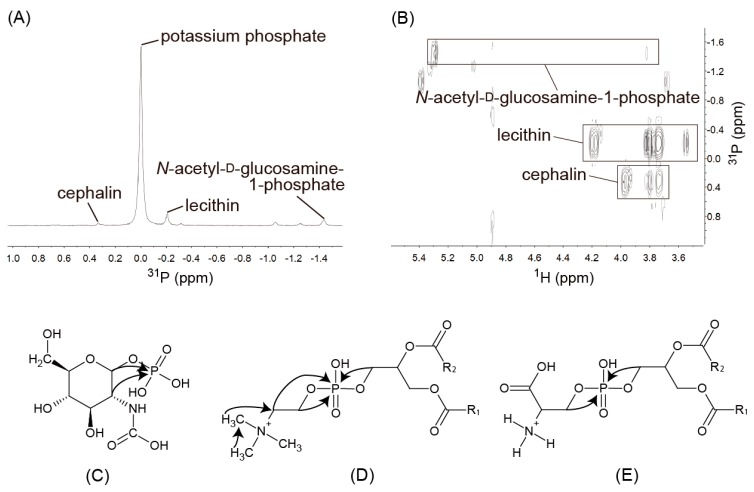
(**A**) The ^31^P NMR spectrum of yogurt. (**B**) The ^1^H-^31^P HMBC NMR spectrum of yogurt. (**C**‒**E**) Correlations of *N*-acetyl-d-glucosamine-1-phosphate (**C**), lecithin (**D**), and cephalin (**E**). Arrows show the cross-peaks in the ^1^H–^31^P HMBC NMR spectrum.

*Lecithin and Cephalin*. In the ^1^H-^13^C HSQC spectrum (Figure 2B), the ^1^H signal at 3.10 ppm was correlated with the ^13^C signal at 54.28 ppm and also showed a correlation with the ^13^C signals at 54.28 and 67.09 ppm in the ^1^H-^13^C HMBC spectrum (Figure 2C). In addition, the ^1^H signal at 3.54 ppm showed a cross-peak with the ^13^C signal at 67.09 ppm. These correlations indicated that yogurt contained compounds with trimethylamine groups. In the ^1^H-^31^P HMBC spectrum (Figure 3B), the ^1^H signals at 3.54 and 4.18 ppm connected with the ^31^P signal at −0.20 ppm supported the existence of trimethylamine groups in yogurt (Figure 3D,E). The ^1^H signals at 3.74 and 3.80 ppm showed correlations with the ^31^P signals at both −0.20 and 0.33 ppm in the ^1^H-^31^P HMBC spectrum, which suggested that the signals were derived from compounds with similar structures. Lecithin and cephalin are the main phospholipids in milk^23^ and have similar structures. Enhancements of the signals in the ^1^H-^31^P HMBC spectrum were observed when standard reagents were added. Therefore, the ^31^P signal at −0.2 ppm that connected the ^1^H signals at 3.55, 3.74, 3.80, and 4.19 ppm was assigned to lecithin, and the ^31^P signal at −0.2 ppm that connected the ^1^H signals at 3.74, 3.80, and 3.97 ppm was assigned to cephalin.

*N-acetyl-d-glucosamine-1-phosphate*. There were some signals that were not assigned in the ^1^H-^31^P HMBC spectrum of yogurt. In a previous study, the signals of *N*-acetyl carbohydrates were detected in whole milk [4]. We hypothesized that the compounds were phosphorylated during fermentation. To assign the remaining signals, we performed spiking experiments with the candidate compounds. The ^1^H-^31^P HMBC spectrum of *N*-acetyl-d-glucosamine-1-phosphate was observed using standard reagents. Based on the ^1^H-^31^P HMBC spectrum (Figure 3B), the ^31^P signal at −1.44 ppm that connected the ^1^H signals at 3.83 and 5.30 ppm was assigned to *N*-acetyl-d-glucosamine-1-phosphate. *N*-acetyl-d-glucosamine-1-phosphate is synthesized from d-glucosamine-1-phosphate, which is one of the products made from lactic acid bacteria and is needed for the synthesis of UDP-α-*N*-acetyl-d-glucosamine-1-phosphate, which is required for UDP-*N*-acetyl-d-glucosamine biosynthesis [21]. UDP-α-*N*-acetyl-d-glucosamine-1-phosphate is an essential precursor of cell wall peptidoglycans, lipopolysaccharides, and enterobacterial common antigens [22].

### 2.4. Concentrations of Yogurt Components

To control the quality and content of components in yogurt, we used a Bulgarian yogurt culture that was prepared by adding a Bulgarian yogurt inoculum into whole milk. After cultivating at 40 °C for 24 h, the sample was applied to NMR measurements for quantification. The components whose signals did not overlap were quantified by the ^1^H NMR spectra, while the other compounds were quantified using the difference spectra that were obtained by subtracting the DOSY spectra from the ^1^H NMR spectra.

**Figure 4:**
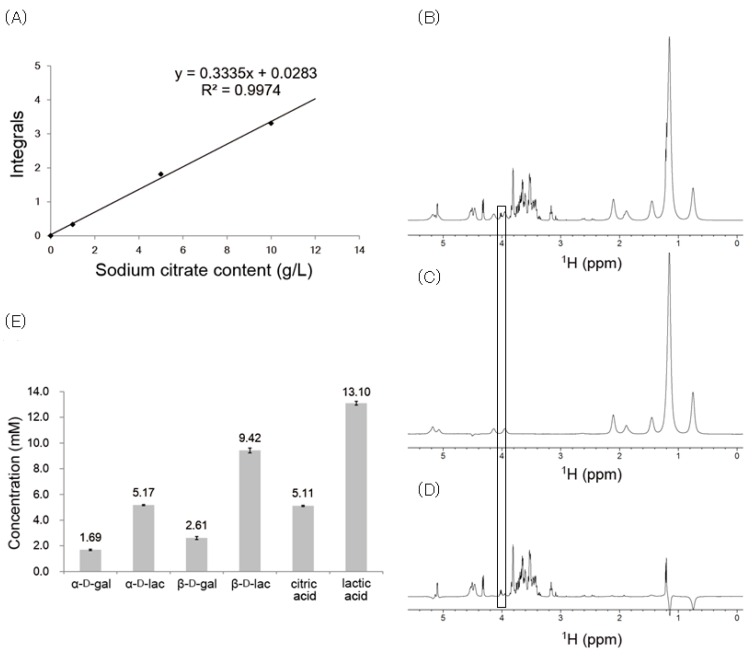
(**A**) Working curves of citrate for the ^1^H NMR spectrum. The integral value of the signal arising from the protons of 1,1,2,2-tetrachloroethane was set to 100. (**B**) Quantitative ^1^H NMR spectrum of yogurt. (**C**) DOSY NMR spectrum of yogurt. (**D**) The difference spectrum for quantifying the integral of the signal at 4.08 ppm between (**B**) and (**C**). (**E**) Concentrations of yogurt components quantified using the ^1^H NMR signals. The standard deviation is shown in parentheses (*n* = 3). Gal and lac are abbreviations for galactose and lactose, respectively.

The ^1^H NMR working curves of the citrate protons are shown in Figure 4A. We used the capillary containing 1,1,2,2-tetrachloroethane for the working curves and quantified the concentrations of the yogurt components. The working curves showed good linearity and therefore were used to calculate the concentrations of yogurt components. In addition, the ^1^H NMR spectra of the self-made yogurt were measured at 40 °C (Figure 4B), and the signals in the difference spectra were used for quantification because the signals were recovered to their original shapes (Figure 4B,D). The inoculum samples were prepared in triplicate with the same fermentation process.

The concentrations of several yogurt components are shown in Figure 4E. The concentrations of α-d-galactose, β-d-galactose, α-d-lactose, β-d-lactose, citric acid, and lactic acid were measured using the quantitative ^1^H NMR spectrum. During milk fermentation, lactic acid bacteria convert part of α-d-lactose and β-d-lactose into α-d-galactose and β-d-galactose, which are finally broken down to become lactic acids, causing a pH decrease that is responsible for casein coagulation [23]. These results showed that, even if the signals in the ^1^H NMR spectrum overlapped with the signals of the acyl chains of fatty acids from milk fats, this quantitative method using quantitative difference spectra can eliminate interference due to overlapping spectra to obtain the correct concentrations of yogurt components.

**Figure 5:**
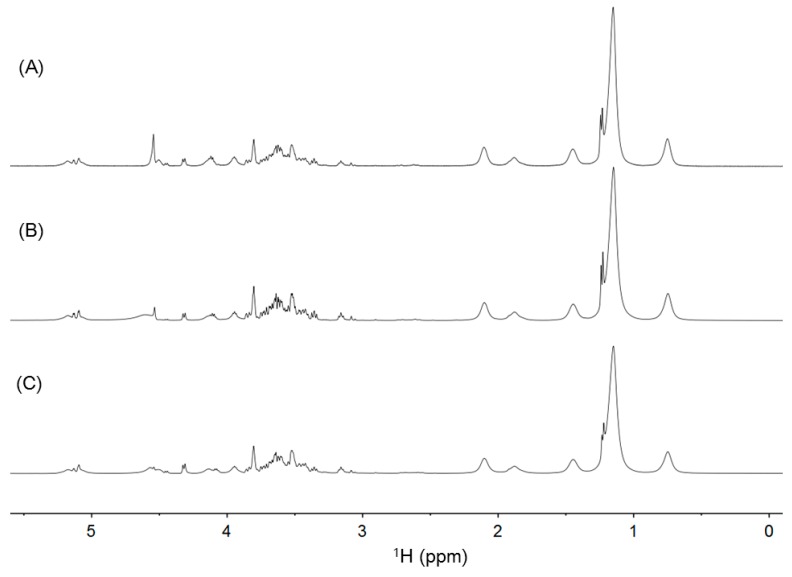
The ^1^H NMR spectra of the ready yogurt measured at 40 °C in different lots.

The ^1^H NMR spectra of the ready yogurt measured at 40 °C in different lots were shown in Figure 5. The spectra and the types of detected components were quite similar among the yogurt in different lots. Both the ready yogurt and the self-made one from inoculum contained two types of bacterial strains: *L. delbrueckii* subsp. *bulgaricus* and *S. thermophiles*. The ^1^H NMR spectra and the NMR-detected components were also similar between the ready yogurt and the self-made one (Figure 4B and Figure 5).

## 3. Experimental Section

### 3.1. Materials and Sample Preparation

Bulgarian yogurt and its inoculum were purchased at a local market. Bulgarian yogurt contained two types of bacterial strains: *L. delbrueckii* subsp. *bulgaricus* and *S. thermophilus*. For signal assignments, D_2_O was added to yogurt at a final concentration of 10% (v/v). Bulgarian yogurt samples (0.6 mL) were then placed in a 5-mm NMR tube (Kanto Chemical Co., Inc., Tokyo, Japan). For quantitative measurements, triplicate yogurt samples were made for this experiment from inoculum. Bulgarian yogurt inoculum (1 g) was added to whole milk (1 L). The sample was immediately mixed with D_2_O at final concentration of 10% (v/v) and was then placed in a 5-mm NMR tube. The volume of the sample was adjusted to 0.6 mL. Fermentation was performed at 40 °C for 24 h after putting the sample into the NMR equipment.

### 3.2. NMR Spectroscopy

NMR experiments were performed at 4 °C on a Unity INOVA-500 spectrometer (Agilent Technologies, Santa Clara, CA, USA) to obtain ^1^H, ^13^C, and ^31^P 1D NMR spectra and ^1^H-^13^C HSQC, ^1^H-^1^H DQF–COSY, ^1^H-^31^P FG-HMBC, and ^1^H-^13^C CT-HMBC spectra.

The ^1^H NMR spectra of yogurt were measured at 499.87 MHz, and the water signal was suppressed by the pre-saturation method. 4,4-dimethyl-4-silapentane-1-sulfonic acid (Wako Pure Chemical Industries, Ltd., Osaka, Japan) was used as an internal reference, and its chemical shift was set to 0 ppm. For assignments, the acquisition parameters were as follows: number of data points, 30,272; spectral width, 8000 Hz; acquisition time, 1.892 s; delay time, 2.000 s; and number of scans, 8. For quantification, NMR experiments were performed at 40 °C, and the acquisition parameters were as follows: number of data points, 32,768; spectral width, 8000 Hz; acquisition time, 2.000 s; delay time, 15.000 s; and number of scans, 32. The free induction decays (FIDs) were zero-filled to 65,536 data points and multiplied by an exponential window function with a 0.25-Hz line-broadening for all ^1^H NMR spectra by NMR software.

The ^13^C NMR spectra were measured at 125.71 MHz. Dioxane was used as an external reference, and its chemical shift was set to 67.5 ppm. The parameters of the ^13^C NMR spectrum were as follows: number of data points, 65,536; spectral width, 31,422 Hz; acquisition time, 1.043 s; delay time, 2.000 s; and number of scans, 83,392.

The ^31^P NMR spectra were measured at 202.35 MHz. Potassium phosphate was used as an external reference, and its ^31^P chemical shift was set to 0 ppm. The ^31^P NMR spectra were measured with the following parameters: number of data points, 30,272; spectral width, 11,999 Hz; acquisition time, 1.043 s; delay time, 2.000 s; and number of scans, 1024. The FIDs were zero-filled to be 65,536 data points and multiplied by an exponential window function with a 0.25-Hz line-broadening for ^31^P NMR spectra by NMR software.

The ^1^H-^1^H DQF-COSY spectra were obtained by suppressing the water signal with the pre-saturation method, and the acquisition parameters were as follows: number of data points, 2048 (F2) and 512 (F1); spectral width, 5911 Hz (F1 and F2); acquisition time, 0.202 s; delay time, 2.000 s; and number of scans, 48.

The ^1^H-^13^C HSQC spectra of yogurt were generated in the phase-sensitive mode with the following acquisition parameters: number of data points, 512 for ^1^H and 256 for ^13^C; spectral widths, 5498 Hz for ^1^H and 20,110 Hz for ^13^C; acquisition time, 0.186 s; delay time, 2.000 s; and number of scans, 80.

The ^1^H-^13^C CT-HMBC spectra were measured in the absolute mode with the following parameters: number of data points, 4096 for ^1^H and 512 for ^13^C; spectral widths, 5498 Hz for ^1^H and 27,643 Hz for ^13^C; acquisition time, 0.402 s; delay time, 3.000 s; and number of scans, 80.

The ^1^H-^31^P CT-HMBC spectra of yogurt were obtained in the absolute mode. Potassium phosphate was used as an external reference, and its ^31^P chemical shift was set to 0 ppm. The acquisition parameters were as follows: number of data points, 2048 for ^1^H and 128 for ^31^P; spectral widths, 5004 Hz for ^1^H and 4047 Hz for ^31^P; acquisition time, 0.402 s; delay time, 1.000 s; and number of scans, 200.

### 3.3. NMR Signal Assignments

The signals in the NMR spectra of yogurt were assigned in reference to the databases, Spectral Database for Organic Compounds (SDBS, http://sdbs.db.aist.go.jp/sdbs) and Biological Magnetic Resonance Data Bank (BMRB Metabolomics, http://www.bmrb.wisc.edu/metabolomics). Because the chemical shifts of several signals were significantly different than those in the published data, their correlations were confirmed in the 2D NMR spectra. Finally, authentic standard compounds were added to yogurt for further confirmation of assignments.

### 3.4. Quantification of Yogurt Components

Because several signals overlapped with signals of the acyl chains of fatty acids from milk fats, quantitative analyses of yogurt were performed using the difference spectrum between the quantitative ^1^H NMR spectrum and the DOSY spectrum. The spin-lattice relaxation times (*T*_1_) for the quantitative ^1^H NMR spectrum were measured by the partial relaxation Fourier-transform (FT) method [4]. The delay time (*d*_1_) was determined with aq being the acquisition time.

$$d_{1}\geq 5\times T_{1}-\text{aq }.$$

A capillary containing 20% (v/v) 1,1,2,2-tetrachloroethane and 80% (v/v) chloroform-d (CDCl_3_) was inserted into the NMR sample tubes as the concentration standard. It has been reported that *T*_1_ of 1,1,2,2-tetrachloroethane was decreased from 2.0 to 0.4 s by adding the relaxation reagent Cr(AcAc)_3_ at a concentration of 1 mg/mL [4]. Therefore, 1 mg/mL Cr(AcAc)_3_ was added to a 20% (v/v) 1,1,2,2-tetrachloroethane solution. The integral value of 1,1,2,2-tetrachloroethane was measured and compared with those of the compounds in yogurt to determine their concentrations.

### 3.5. Diffusion Ordered Spectroscopy (DOSY)

DOSY separates individual components in mixtures based on their diffusion. Molecules in solution are in constant motion and are accompanied by both rotational and translational motions. Diffusion experiments were performed in the *z*-direction using the DgcsteSL_dpfgse sequence [24]. The acquisition parameters were as follows: number of data points, 32,768; spectral width, 8000 Hz; acquisition time, 2.048 s; delay time, 15.000 s; number of scans, 16; diffusion delay, 0.400 s; total diffusion-encoding gradient pulse duration, 0.002 s; gradient stabilization delay, 0.0003 s. The signals that overlapped with those of milk fats were quantified using difference spectra that were obtained by subtracting the DOSY spectra from the quantitative ^1^H NMR spectra.

## 4. Conclusions

In conclusion, ^1^H, ^13^C, and ^31^P NMR spectra, as well as ^1^H-^13^C HSQC, ^1^H-^1^H DQF-COSY, ^1^H-^31^P CT-HMBC, and ^1^H-^13^C CT-HMBC spectra, of yogurt were successfully obtained without any separation or pretreatment. In addition, the quantification of yogurt components was conducted using the ^1^H NMR spectra and the difference spectra between the quantitative ^1^H NMR spectra and the DOSY spectra. Therefore, this study of yogurt using NMR spectroscopy provides a promising method to monitor the various components produced during yogurt fermentation and to classify yogurt types. Moreover, the assignment data and quantitative method can be utilized for quality control and other applications. The proposed method could be useful to analyze the differences between various kinds of yogurt.

## Figures and Tables

**Table 1:** Assignment of ^1^H and ^13^C signals of compounds in yogurt.

Compound	^1^H Multiplicity J (Hz)	Chemical Shift (ppm)			Assignment ^a^
		^1^H	^13^C	^31^P	
d-lactose	d(7.70)	4.33	103.57		CH-1′
		3.43	71.79		CH-2′
		3.54	73.31		CH-3′
		3.80	69.38		CH-4′
		3.61	76.15		CH-5′
		3.64	61.94		CH_2_OH-6′
	d(3.05)	5.11	92.62		CH-α1
	t(8.85)	3.47	71.98		CH-α2
		3.71	72.24		CH-α3
		3.54	78.89		CH-α4
		3.82	70.86		CH-α5
		3.76	60.66		CH_2_OH-α6
	d(7.90)	4.55	96.55		CH-β1
	t(7.83)	3.16	74.66		CH-β2
		3.53	75.17		CH-β3
		3.54	78.75		CH-β4
		3.52	75.56		CH-β5
		3.82	60.80		CH_2_OH-β6
d-galactose	d(3.35)	5.14	93.06		CH-α1
		3.69	69.18		CH-α2
		3.72	69.99		CH-α3
		3.85	70.11		CH-α4
		3.96	71.25		CH-α5
		3.66	62.02		CH_2_OH-α6
	d(7.80)	4.46	97.23		CH-β1
		3.36	72.70		CH-β2
		3.53	75.17		CH-β3
		3.82	69.56		CH-β4
		3.60	75.94		CH-β5
		3.63	61.84		CH_2_OH-β6
acyl chains of fatty acids		0.76	14.74		CH_3-_ω1
		1.17	23.54		CH_2_-ω2
		1.45	25.60		CH_2_-Δ3
		1.89	27.93		oleate CH_2-_ω8,11
		1.17	29.78–30.90		CH_2_(ω4-n)
		1.14	32.79		CH_2_-ω3
		2.11	34.37		CH_2_-Δ2
		5.19	130.18		oleate HC=CH
glycerol backbone of fats		3.98, 4.16	62.54		glycerol-1,3
		5.10	69.59		glycerol-2
acetic acid	s	1.93	22.72		CH_3_
			175.40		COOH
citric acid	AB	2.53,2.69	44.93		CH_2_
			75.80		C
			177.70		CH_2_-COOH
			181.40		COOH
lactic acid	d(6.60)	1.23	20.84		CH_3_
	dd	4.08	68.95		CH
			182.50		COOH
alanine	d(7.15)	1.36	17.09		CH_3_
		3.66	51.49		CH
			176.4		COOH
creatine	s	2.90	37.40		CH_3_
	s	3.81	54.78		CH_2_
			158.30		C
			175.40		COOH
lecithin	s	3.10	54.28		trimethylamine
		3.74, 3.80		−0.20	CH_2_-3
		4.18	60.22	−0.20	CH_2_-4
		3.54	67.09	−0.20	CH_2_-5
cephalin		3.74, 3.80		0.33	CH_2_-3
		3.97		0.33	CH_2_-4
*N*-acetyl-d-glucosamine-1-phosphate		5.30		−1.44	*O*-CH
		3.83		−1.44	*N*-CH

^a^ The symbol “ω” indicates the position from the methyl group; the symbol “Δ” indicates the position from the ester group.
